# Adipose stem cells in obesity: challenges and opportunities

**DOI:** 10.1042/BSR20194076

**Published:** 2020-06-09

**Authors:** Sunhye Shin, Asma S. El-Sabbagh, Brandon E. Lukas, Skylar J. Tanneberger, Yuwei Jiang

**Affiliations:** Department of Physiology and Biophysics, College of Medicine, The University of Illinois at Chicago, IL 60612, U.S.A.

**Keywords:** adipose tissue development, APCs, ASCs, homeostasis, obesity, thermogenesis

## Abstract

Adipose tissue, the storage of excessive energy in the body, secretes various proteins called adipokines, which connect the body’s nutritional status to the regulation of energy balance. Obesity triggers alterations of quantity and quality of various types of cells that reside in adipose tissue, including adipose stem cells (ASCs; referred to as adipose-derived stem/stromal cells *in vitro*). These alterations in the functionalities and properties of ASCs impair adipose tissue remodeling and adipose tissue function, which induces low-grade systemic inflammation, progressive insulin resistance, and other metabolic disorders. In contrast, the ability of ASCs to recruit new adipocytes when faced with caloric excess leads to healthy adipose tissue expansion, associated with lower amounts of inflammation, fibrosis, and insulin resistance. This review focuses on recent advances in our understanding of the identity of ASCs and their roles in adipose tissue development, homeostasis, expansion, and thermogenesis, and how these roles go awry in obesity. A better understanding of the biology of ASCs and their adipogenesis may lead to novel therapeutic targets for obesity and metabolic disease.

## Introduction

Obesity is on the rise, tripling in prevalence from 1975 to 2016 and creating a global epidemic. Recent statistics show that 39% of adults (18 years or older) are overweight (with a body mass index (BMI) greater than or equal to 25 kg/m^2^) and 13% are obese (with BMI greater than or equal to 30 kg/m^2^) [1]. In addition, 340 million children and adolescents, aged 5–19, are overweight or obese (as of 2016) [1]. By 2030, 50% of the global population is expected to be overweight or obese [2]. Obesity contributes to the aging process and can increase the risk of various other health problems, including insulin resistance, dyslipidemia, atherosclerosis, hypertension, and certain types of cancer [3]. According to prospective studies, there is a 30% increase in general mortality rate for each increase of 5 kg/m^2^ in BMI among individuals with a BMI above 25 kg/m^2^ [4]. On the other hand, men with a BMI between 23.5 and 24.9 kg/m^2^ and women with a BMI between 22.0 and 23.4 kg/m^2^ have the lowest mortality rate [5].

Adipose tissue is central to the control of energy balance and lipid homeostasis and therefore at the nexus of the obesity epidemic. Adipose tissue comprises adipocytes, endothelial cells, fibroblasts, immune cells, and adipose stem cells (ASCs). ASCs (also referred to as adipose-derived stem/stromal cells *in vitro*) are able to self-divide and differentiate into new adipocytes. In addition to adipose tissue development and homeostasis, several studies have suggested that ASCs play a critical role in the development of obesity and obesity-related metabolic diseases [6–9]. Furthermore, the dysfunction of adipocytes in the obese state can also alter the numbers and the functions of ASCs [10], leading to abnormal adipose tissue remodeling associated with a higher risk of metabolic disorders [3]. In contrast with white adipose tissues (WATs) from patients with metabolic syndrome, WATs from metabolically healthy individuals display relatively smaller adipocytes with greater blood vessel density. Recent compelling studies suggest that new adipogenesis derived from ASCs may produce healthy adipose tissue expansion [11–13]. Therefore, understanding the function and contribution of ASCs to the progression of obesity is fundamental to developing new therapeutic approaches that prevent or treat obesity and its complications.

There are three distinct types of adipocytes: white, brown, and beige, and they have different physiological roles in energy metabolism. While white adipocytes store surplus energy as triacylglycerol (TAG), brown and beige adipocytes act as cellular furnaces that burn blood glucose and fatty acids to generate heat [14–16]. Unlike brown adipocytes that have a discrete anatomic location and express high levels of thermogenic genes under basal conditions, beige adipocytes are recruited within WATs and express thermogenic genes after stimuli such as cold exposure or β-adrenergic stimulation [14,15]. Evidence suggests that these recruited beige adipocytes within WATs are developmentally, molecularly, and functionally distinct from classical brown adipocytes [17–19]. Various genetic mouse models have suggested that the ability to recruit beige adipocytes within WAT has beneficial effects not only on body fat, but also on metabolic health [20–25]. Recent studies indicate that beige adipocytes are present in adult humans, and their activities are correlated with a metabolically healthy phenotype [26–28]. This metabolic correlation signifies that the ASCs can be manipulated to generate beige fat to counteract obesity and diabetes. However, the recruitment of new beige adipocytes collapses in obesity, creating a key challenge to its therapeutic promise. It remains poorly understood about the underlying mechanism(s) by which obesity impedes beiging potential and how to counteract that and rejuvenate beiging potential. In this review, we discuss the characterization of ASCs, the role of ASCs in different types of adipose tissue, the relationship between ASCs and metabolic health in obesity. In addition, we further explore related strategies that involve using ASCs to prevent and treat obesity and obesity-related diseases.

## Distinct types of adipose tissue

### WAT

WAT is composed of unilocular adipocytes, containing a single large lipid droplet (LD), whose primary function is to store surplus energy in the form of TAG [18]. Adipocytes are also active endocrine cells that secrete adipokines, such as leptin and adiponectin, which regulate metabolism by maintaining insulin sensitivity and consuming glucose [29]. There are generally two main categories of WAT: subcutaneous adipose tissue (SAT) attached to the dermal layer and visceral adipose tissue (VAT) which accumulates near other organs inside the intra-abdominal cavity [30]. SAT is considered to be relatively beneficial since it serves as a protective cushion against physical impact as well as a thermoregulating insulator against cold. By contrast, VAT is potentially harmful since visceral fat depots have higher lipolytic responses and releases free fatty acids more readily than SAT [31]. Due to its proximity to the liver, secreted free fatty acids from visceral fat depots can exert adverse effects on the liver through the portal vein, contributing to the development of metabolic diseases, including dyslipidemia, insulin resistance, atherosclerosis, and hypertension [32].

The metabolic roles and distributions of SAT and VAT greatly vary, and their distinct characteristics are preserved despite transplantation, indicating SAT and VAT may emerge from distinct progenitor cells [30]. Studies on murine adipose tissue organogenesis have shown that SAT and VAT are distinguishable in terms of the developmental timing as well. SAT depots, including inguinal and interscapular WAT, are developed during embryogenesis, whereas VAT depots, including mesenteric and retroperitoneal WAT, are formed after birth [33,34]. However, patterns of adipogenesis differ between species. In rodents, most WAT depots are formed postnatally and the adipocytes are renewed at a rate of 0.6% each day [35]. In humans, adipocytes mostly form early in post-natal periods, and continue to expand in size as they accumulate fat in the course of childhood. The next stage of new adipocyte formation sets in during puberty, and the cells proliferate in bodily distributions that are maintained throughout adulthood with approximately 8% variation and regeneration every year [35].

### Brown and beige adipose tissue

Brown adipose tissue (BAT) is also an endocrine organ like WAT, consisting of brown adipocytes and other cell types, and secretes different cytokines, hormones, and other factors. However, unlike white adipocytes which contain a single large LD and are more efficient for energy storage [36], brown adipocytes are highly vascularized and filled with multiple small LDs and mitochondria. Through uncoupling protein 1 (UCP1), brown adipocytes facilitate heat generation from the stored energy [37].

Beige adipocytes are multilocular UCP1-positive adipocytes which exist in WAT depots. They act like white adipocytes in basal conditions but become brown-like cells in response to various stimuli including cold exposure and catecholamine administration. Thus, they increase energy expenditure by inducing thermogenesis [36]. Chronic cold exposure or β-adrenergic stimulation promotes the acquisition of a BAT-like phenotype of WAT by enhancing the appearance of multilocular UCP1-positive adipocytes [38]. After the stimulation, metabolic rate, fatty acid oxidation, and mitochondrial biogenesis are increased [39]. Also, the size of LDs is gradually decreased, and the number of small- or micro-LDs increases due to the lipolysis [36].

While beige and brown adipocytes express common thermogenic genes, they are different in gene expression profiles and developmental origins. Whereas brown adipocytes are derived from myogenic factor 5 encoding gene (*Myf5*+) cells, beige adipocytes share the same lineage, which is *Myf5*− cells, as white adipocytes [14,40]. Cluster of differentiation (CD) 137 (CD137) and TMEM26 are highly expressed in beige adipocytes compared with brown adipocytes [41]. In addition, brown adipocytes have high levels of UCP1 and other thermogenic genes even under basal conditions; however, beige adipocytes express these genes only when treated with activators of the β-adrenergic receptors (β-AR) or peroxisome proliferator-activated receptor (PPAR) γ (PPARγ) [42,43].

## Adipose tissues in obesity

### Adipose tissue expansion in obesity

In the presence of excess caloric intake, both SAT and VAT expand, which is considered to be developed as an evolutionary mechanism for fat storage [29]. WAT expansion is achieved through hypertrophy (cell size increase) and hyperplasia (cell number increase) of adipocytes [44]. The latter process exemplifies *de novo* adipocyte formation (adipogenesis). In obesity, WAT may fail to appropriately expand to store surplus energy, which leads to ectopic fat deposition in other tissues such as the liver, skeletal muscle, and the pancreas. At the whole-body level, this results in progressive insulin resistance and an increased risk of type 2 diabetes.

While adipose tissue expansion is mediated by adipocyte hyperplasia in childhood obesity, it is considered that adipose tissue mass is increased primarily by adipocyte hypertrophy in adult obesity. This idea is supported by the previous studies showing that lean and obese adults have roughly similar numbers of adipocytes [45], and obesity does not alter the number or the turnover rate of adipocytes in adults [46]. Additionally, adult mice were shown to have lower adipogenic potential under high-fat diet (HFD) feeding compared with juvenile mice, indicating that decreased generation of new adipocytes with advancing age could contribute to metabolic failure [47].

Although obesity drives WAT expansion through both hypertrophy and hyperplasia, evidence suggests that SAT and VAT undergo different rates of adipogenesis during development and expansion [33]. Mouse VAT sustains high adipogenic rate during HFD exposure, while SAT maintains a low rate of adipogenesis [33]. Human data suggest that overfeeding induces adipocyte hypertrophy in upper SAT, but hyperplasia in lower parts [48]. It is also important to note that SAT and VAT play contrasting roles when it comes to obesity both in human subjects and mouse models. Transplantation of SAT into VAT has been shown to suppress body weight gain and ameliorate insulin tolerance and inflammation [49,50]. Also, the metabolic functions of obese mice are closer to normal when hyperplasia occurs in the subcutaneous region rather than in visceral fat [12]. In contrast, in the VAT of obese mice, hypertrophic adipocytes contribute to tissue inflammation by secreting elevated levels of cytokines [30]. The higher waist-to-hip ratio and abdominal diameter that indicate upper-body or visceral obesity are related to the higher plasma glucose, insulin, and TAG levels, the higher blood pressure, and the lower high-density lipoprotein (HDL) cholesterol levels than lower-body or subcutaneous obesity [51].

### Adipose tissue inflammation and thermogenesis in obesity

Obesity stimulates quantitative and qualitative changes in various types of leukocytes residing in adipose tissue, and this change elevates expression levels of inflammatory cytokines and adipokines [52]. Adipose tissue macrophages (ATMs) form 10–15% of the stromal vascular fraction (SVF) of adipose tissue in lean state, and approximately 50% of SVF in obese state [53]. ATMs are found to regulate not only inflammatory responses in adipose tissue, but also thermogenic remodeling of adipose tissue [10]. Among two types of ATMs, type 1 macrophages (M1) are classically activated macrophages which secrete pro-inflammatory cytokines, such as tumor necrosis factor-α (TNF-α) and interleukin (IL) 6, and generate reactive oxygen species (ROS) by activating inducible nitric oxide synthase (iNOS). Type 2 macrophages (M2) are alternatively activated macrophages that secrete anti-inflammatory markers, such as IL-10 and arginase which block iNOS activity [54].

Widely observed in healthy adipose tissue, M2 ATMs control tissue homeostasis [54]. M2 ATMs express not only anti-inflammatory cytokines but also catecholamines which activate beige adipocytes by stimulating β-adrenergic signaling in WAT [55,56]. When M2 ATMs were depleted, thermogenic gene expression, lipolysis, and energy expenditure were not increased after cold exposure. Administration of IL-4, which activates M2 ATMs, increased thermogenic gene expression, lipolysis, and energy expenditure [57]. Interestingly, it was also reported that M2 ATMs, through β-adrenergic signaling, promote recruitment of platelet-derived growth factor receptor (PDGFR) α (PDGFRα)-expressing ASCs that differentiate into beige adipocytes in WAT during cold acclimation [57].

However, obesity induces ATM polarization from anti-inflammatory M2 to pro-inflammatory M1 state. This change causes inflammation and induces infiltration of more M1 ATMs by monocyte chemoattractant protein-1 (MCP-1). Infiltrated M1 ATMs locate around dead adipocytes and form crown-like structures (CLS), which further up-regulates pro-inflammatory cytokine secretion [53,54,58]. In the context of obesity, IL-4 expression and secretion are down-regulated [59], and suppression of IL-4 signaling suppresses beige adipogenesis in SAT and decreases whole body thermogenesis [57].

Although hyperplasia of adipocytes is considered to be a healthy expansion of WAT, hypertrophy of adipocytes could lead to multiple metabolic disorders [10]. Hypertrophic adipocytes undergo necrotic-like death in obesity [60], and increased expression and secretion of pro-inflammatory cytokines, including TNF-α, IL-6, IL-8, and MCP-1. These cytokines recruit various immune cells into adipose tissue, which causes inflammation [61]. Hypertrophic adipocytes also go through hypoxia, and hypoxic responses mediated by hypoxia-inducible factor (HIF) 1α and HIF-2α induce adipose fibrosis and inflammation [62]. Taken together, these traits of hypertrophic adipocytes result in malfunction of adipocytes and ASCs.

## Overview of ASCs in physiology and disease

### ASCs characterization *in vitro* and *in vivo*

ASCs, first isolated in the 1960s, are stem cells found within the SVF component of adipose tissue [63,64]. As a multipotent mesenchymal stem cell, ASCs have the potential to differentiate into multiple cell types, including fibroblasts, myoblasts, cardiomyocytes, chondrocytes, osteoblasts, and adipocytes [29,63]. In the presence of insulin-like growth factor-1, glucocorticoids, and other adipogenic stimuli, ASCs gain the potential to differentiate into adipocytes [30]. Adipose progenitor/precursor cells (APCs) are early descendants of ASCs that are committed to differentiate into adipose lineage. While ASCs are multipotent and can self-renew, APCs can only divide for a limited number of times and are specialized to differentiate specifically into adipocytes [65,66]. Both ASCs and APCs are found in the SVF component of adipose tissue, and these two distinct cell populations can be separated by flow cytometry analysis using antibodies against specific cell surface markers. A recent single-cell RNA sequencing data have revealed that the expression level of CD55 is high in ASCs but low in APCs [66]. CD55^high^ populations were shown to have significantly faster proliferation but less differentiation capacities than CD55^low^ populations [66].

In the early adipogenic differentiation process, APCs increase the expression of CCAAT/enhancer binding protein (C/EBP) β after a growth arrest state [67]. In these committed white fat progenitor cells, expression of C/EBPα and PPARγ is up-regulated [68], which induces adipocyte differentiation. During the later differentiation phase, the cells undergo extreme changes in cell shape, developing a spherical shape to fully differentiated adipocytes. At the terminal differentiation stage, C/EBPα and PPARγ target gene expression levels are extremely increased [69]. Since the downstream target genes include lipogenic enzymes such as acetyl-CoA carboxylase (ACC) and fatty acid synthase (FAS), the up-regulation of C/EBPα and PPARγ promotes lipid accumulation in adipocytes [70].

The roles of C/EBPα and PPARγ in adipogenesis are essential. PPARγ is the most important transcription factor for adipogenesis [71], and C/EBPα induces and maintains PPARγ expression, and regulates insulin-related genes [72]. Therefore, differentiated adipocytes become insulin-sensitive by increasing the expression of glucose transporters (GLUTs) and insulin receptors [71]. Since C/EBPα and PPARγ regulate the expression of each other [71], deletion of either gene leads to the disruption of adipose tissue development [73,74]. On the other hand, overexpression of either gene is sufficient to induce adipogenic differentiation of non-adipogenic fibroblasts [75–77].

Recent studies have identified multiple cell surface makers for ASC identification (Table 1). It is determined that the minimal phenotypic criteria to characterize ASCs are cells expressing surface markers such as the CD44, CD73, and CD90, but not CD45 or CD31 [78]. Other ASC surface markers, such as CD34, may change expression with division, which creates different ASC subpopulations [78]. ASC markers differ with respect to where and when the stem cell was formed [29]. For instance, Myf5-Cre marks ASCs in interscapular and retroperitoneal but not inguinal or perigonadal fat depots, and SMA-Cre marks adult but not developmental ASCs [29,34]. Future studies identifying more specific ASC markers should be beneficial as they may lead to the creation of genetic tools designed to manipulate adipose tissue, control adiposity, and combat diabesity [29].

**Table 1:** Cell surface markers of ASCs

	Markers	References
Positive	CD24	[9,149–152]
	CD29	[149,151,153]
	CD34	[78,80,85,86,149,151–154]
	CD44	[78,154]
	CD73	[78]
	CD90	[78]
	Decorin	[154]
	gp38	[152]
	NG2	[80]
	PDGFRα	[85,86,151,152,155]
	PDGFRβ	[79,80,86]
	Sca1	[80,85,86,149,151–153,156]
	VCAM1	[152]
Negative	CD31	[78,80,149,151–154]
	CD45	[78,80,149,151–154]
	CD105	[80,149]
	CD117	[149,154]
	Mac1	[80]
	Ter119	[80,149]

### Lineage tracing to study adipogenesis *in vivo*

**Figure 1:**
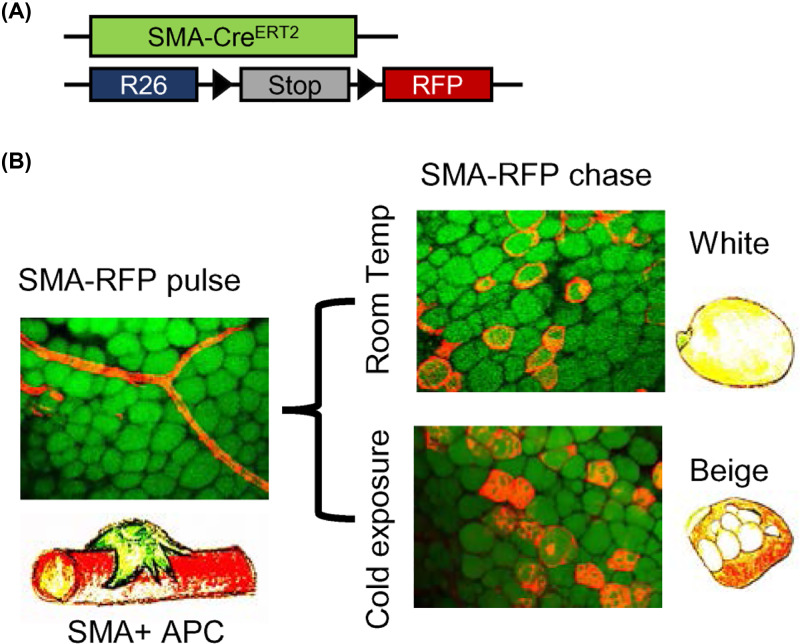
Tracking adipogenesis using Mural-Trak (SMA+) fate mapping model (**A**) Genetic alleles for tracing SMA cells. (**B**) SMA+ APCs at pulse (2 days after tamoxifen) or at chase at room temperature or cold challenged. SMA+ labeled perivascular cells can give rise to both white and beige adipocytes.

Although we can address many cellular and molecular aspects of adipogenesis using adipocyte cell culture models, a large number of questions have not been answered, including the developmental origins and their exact location of ASCs within the SVF of adipose tissue. During the last few decades, researchers have begun to study adipogenesis using *in vivo* lineage tracing models (Box 1). Tracking of ASCs *in vivo* has revealed that adult ASCs reside near and along the blood vessels of adipose tissue [29]. Adult ASCs resemble mural cells in that they express several mural cell markers such as PDGFRβ and α smooth muscle actin (α-SMA), further supporting the notion of a vascular residency [29]. Multiple ASC lineage markers have been identified, several of which can be used in genetic models to mark adipocytes in specific phases of adipose tissue biology (Table 2). For instance, our group has recently used an SMA+ fate mapping tool in which the SMA promoter drives Cre expression (Figure 1). Using this system, we found that SMA+ cells generate both white and beige adipocytes during adipose tissue homeostasis and thermogenic phases, but not the developmental phase [34,79]. When PPARγ, a master regulator of adipogenesis, was deleted in SMA+ cells at postnatal day (P) 10, adipocyte growth and development yielded normal results. However, deletion of PPARγ in SMA+ cells at P30 significantly reduced fat depot size, modified the molecular profile of adipocytes, and affected glucose tolerance [34]. This suggests that adipocytes used to replenish cellular turnover in adults arise from SMA+ precursors, and that SMA+ precursors are responsible for the adult AT homeostasis [79].

Box 1 **Cell culture vs. lineage tracing approaches to study adipogenesis**

Cell culture and lineage tracing approaches are two distinct methods which researchers use to study ASCs. Cell culture techniques enable researchers to expand, maintain, and study ASCs in a controlled environment [146], and lineage tracing approaches genetically label cells and their progeny, thus enabling researchers to study the renewal, fate behavior, proliferative hierarchy, and lineage potential of ASCs in embryonic, postnatal, and adult tissue [147]. While *in vitro* cell culture and *in vivo* lineage tracing are both commonly used techniques to study ASCs, data from these two approaches are inconsistent. Whereas ASCs from SAT are more adipogenic than those from VAT under *in vitro* cell culture conditions [148], *in vivo* lineage tracing systems revealed that ASCs from obese SAT do not rapidly proliferate and differentiate into adipocytes compared with those from obese VAT [9]. The inconsistency between the two methods leads scientists to speculate that the different adipogenic potentials of SAT and VAT are reflective of specific tissue environmental cues and differences in the intrinsic characteristics of ASCs from each fat depot [30]. Therefore, while many parameters can be controlled with a cell culturing approach, its serving as a physiologically relevant model poses a potential challenge [146].

**Table 2:** Mouse strains for adipose lineage tracing

Lineage	AT depot marked	Phase of AT biology	References
Adiponectin-rtTA	SAT, VAT	Development, Homeostasis, Expansion, Thermogenesis	[33]
aP2-Cre	SAT, VAT	Development, Homeostasis	[80,81]
En1-Cre^ER^	BAT	Development	[157]
HoxB6-Cre^ER^	Inguinal WAT, mesenteric WAT	Homeostasis	[158]
Lysm-Cre	perigonadal WAT	Not determined	[87]
Meox1-Cre	BAT, retroperitoneal WAT, interscapular WAT	Not determined	[158]
Myf5-Cre	BAT, SAT, retroperitoneal WAT	Not determined	[89,159]
Myh11-Cre^ER^	Not determined	Thermogenesis	[160]
NG2-Cre^ER^	SAT	Thermogenesis	[79]
Pax3-Cre	BAT, SAT, retroperitoneal WAT, perigonadal WAT	Not determined	[89]
Pax7-Cre	BAT, interscapular WAT	Not determined	[158]
Pax7-Cre^ER^	BAT	Development	[161]
PDGFRα-Cre^ER^	VAT	Expansion, Thermogenesis	[85]
PDGFRβ-rtTA	Inguinal WAT, perigonadal WAT	Expansion, Thermogenesis	[86]
PPARγ-tTA	SAT, VAT	Development, Homeostasis, Expansion, Thermogenesis	[34,79,80]
Pref1-rtTA	SAT, VAT	Development, Homeostasis	[82]
Prx1-Cre	SAT	Not determined	[90]
SM22-Cre	Inguinal WAT, perigonadal WAT	Thermogenesis	[79,80]
SMA-Cre^ER^	SAT, VAT	Homeostasis, Thermogenesis	[34,79]
Sox10-Cre	WAT around salivary gland and ear	Not determined	[88]
VE-Cadherin-Cre	SAT, VAT	Development, Homeostasis	[84]
WT1-Cre	VAT	Development, Homeostasis	[83]

SM22-Cre is another lineage marker which is not present during the developmental phase as well [79,80]. On the other hand, aP2 (adipocyte protein 2)-Cre, Pref-1 (preadipocyte factor 1)-rtTA, WT1 (Wilms tumor gene)-Cre, and Vascular endothelial (VE)-Cadherin-Cre were reported to mark adipocytes in both the developmental and homeostatic phases [80–84]. PDGFRα-Cre^ER^ and PDGFRβ-rtTA, mark adipocytes in both the expansion and thermogenic phases, and adiponectin-rtTA and PPARγ-tTA are found in all phases of adipose tissue biology [33,34,79,80,85,86].

In addition to marking adipocytes during specific phases of adipose tissue biology, multiple ASC lineage markers may be used to target specific WAT depots. For example, Lysm (lysin motif)-Cre marks adipocytes in bone marrow WAT, and Sox10 (SRY-Box transcription factor 10)-Cre marks those in head-neck WAT [87,88]. Some ASC lineage markers, such as Myf5-Cre and Pax3 (paired box gene 3)-Cre, mark adipocytes in interscapular SAT and retroperitoneal VAT while others, like SM22 (smooth muscle protein 22)-Cre and PDGFRβ-rtTA, mark adipocytes in inguinal SAT and perigonadal VAT [79,80,86,89]. Prx-1 (Paired-related homeobox 1)-Cre marks adipocytes in all of SAT only (inguinal and interscapular) whereas both aP2-Cre and adiponectin-rtTA mark adipocytes in all of SAT and VAT [33,80,81,90]. Identification of more adipose stem cell lineage markers will be useful for tracking and manipulating adipose tissue, especially during specific phases and in specific depots.

### Heterogeneity of ASCs between WAT depots

ASCs with depot-specific gene expression profiles and adipogenic potentials are keys for the establishment of the functional heterogeneity between WAT depots, as well as for the development of depot-specific responses to metabolic challenges. For example, a subset of ASCs express PDGFRα, which is a receptor found in the precursor cells of both SAT and VAT [29]. However, more recent studies shed light on the effects of positive or negative expression of PDGFRα on the ontogeny of SAT and VAT, and on the heterogeneous lineages that give rise to each type of tissues. In SAT, all ASCs express PDGFRα and can differentiate into either white or beige adipocytes. However, not all ASCs in VAT positively express PDGFRα, and PDGFRα− precursors can only produce white and unilocular adipocytes with few mitochondria [91]. These findings help to explain the pathophysiological characteristics of VAT that may stem from the abundance of its PDGFRα negative white adipocytes, which do not exist in the morphology of SAT. Furthermore, the homogeneity of ASCs of SAT in healthy *ex vivo* human tissue has been affirmed through single-cell RNA sequencing [92].

Recent advances in lineage tracing studies have identified a number of ASC/APC populations with distinct markers. It remains unclear what degree of heterogeneity exists among APCs. For example, fate mapping data from SMA+ cells do not allow us to discriminate whether functionally distinct SMA+ APCs exist to give rise to white and brown adipocytes, or if there is a common progenitor for all adipocytes in different depots. With the rapid development of single-cell RNA sequencing, distinct subpopulations of APCs in the SVF of WAT are seen to present in both mouse and human adipose tissues [93–98]. It will be of future interest to study these APC subpopulations and their contributions to adipogenesis in physiology and obesity along with depot-specific manner.

### Tracking ASCs in obesity

Obesity elevates expression levels of pro-inflammatory cytokines and adipokines by stimulating quantitative and qualitative changes in various types of leukocytes [52], and this obesity-induced chronic inflammation significantly impairs ASC function [63]. For example, ASCs isolated from an obese environment have impaired differentiation and migration properties [99–102]. Other studies show that ASCs isolated from obese patients have alterations in their transcriptomic profile that indicate loss in ‘stemness’ phenotype [103]. Remarkably, the detrimental state of ASCs triggered by an obese environment is only partially reversed after weight loss, largely due to the accumulation of epigenetic modifications in ASCs [104,105]. While non-obese ASCs are insulin sensitive and show suppressed basal lipolysis levels, obese-derived ASCs are insulin resistant, resulting in impaired glucose uptake and increased lipolysis [99]. In addition, ASCs from obese mammals have an increased number of mitochondria that do not function well [106], and have higher levels of ROS than those from non-obese mammals [105]. Obese-derived ASCs display specific differences with regard to metabolic regulators and modulators, including vitamin D, Gas5 (Growth arrest-specific 5; a non-coding RNA involved in glucocorticoid resistance) [107], and sirtuins (a family of proteins modulating adipose tissue metabolism) [108]. They also have higher levels of metabolites related to carbohydrate metabolism [109]. Interestingly, cytosolic transfer from healthy ASCs to obese-derived ASCs was shown to restore insulin sensitivity. It is considered that the transfer restores the Lin28/Let 7 pathway, involved in a feedback loop with nuclear factor-κB (NF-κB) and TNF-α, confirming that there is a link between obesity-induced inflammation and ASC cellular insulin resistance [99]. Collectively, these results demonstrate that obesity decreases the number of healthy and functioning ASCs which are necessary for adipose tissue metabolism.

**Figure 2:**
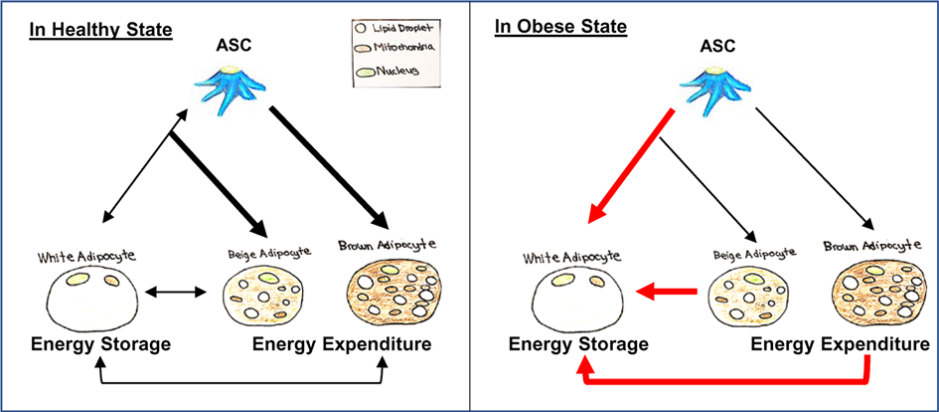
Schematic diagram of adipogenesis in healthy and obese states ASCs give rise to different adipocytes. White adipocytes store energy, whereas brown and beige adipocytes are specialized for energy expenditure. Of note, transdifferentiation between beige and white adipocytes, or between brown and white adipocytes are also suggested. In addition, mature white adipocytes have the ability to dedifferentiate into multipotent ASCs. In obese state, the roles of ASCs get altered, leading to the generation of more white fat and the whitening of thermogenic brown and beige fat.

Obesity drives the pronounced ‘whitening’ of beige and BAT. In addition, it is reported that positive energy balance provokes the proliferation of ASCs, and when adipocytes reach a volume limit, the newly formed ASCs are utilized for *de novo* adipogenesis to further increase energy storage capacity of adipose tissue [9,33] (Figure 2). Among various ASCs, PDGFRα+ and PDGFRβ+ cells were found to proliferate under HFD feeding. PDGFRα-Cre^ER^ labeled 25% of the abdominal fat in obese mice [85], and PDGFRβ-rtTA contributed to mark expanded WAT [86]. These data indicate that PDGFRα and PDGFRβ expressing ASCs are biologically involved in WAT expansion [85,86]. Additional research is still required to uncover the mechanism that governs how ASCs generate new adipocytes in obesity, and the impact of environmental and genetic factors on this response.

## Therapeutic applications of ASCs

### Challenges and opportunities

ASCs have received plenty of attention for their therapeutic potential. As multipotent stem cells, ASCs can differentiate into various types of cells, including adipocytes, osteocytes, chondrocytes, and myocytes, and also exert immunomodulatory effects [110]. Adipose tissues are known to yield 100–500-times higher number of stem cells compared with bone marrow, and harvesting adipose tissue through liposuction is less risky [111,112]. Therefore, ASC therapy is considered to be very promising for diseases with limited therapeutic options.

ASCs are able to regenerate multiple tissues and improve their functions. Human ASCs improved myocardial function in mice with myocardial infarction. Some of the injected cells were shown to differentiate into cardiomyocytes and others were used for formation of new vessels [113]. A direct intramyocardial injection of ASCs improved heart function and tissue viability, increased angiogenesis, and decreased fibrosis in rats with left coronary artery ligation [114]. In pigs whose left anterior descending coronary artery was blocked, delivery of ASCs was shown to reduce scar volume of the left ventricle wall and to increase mass of the left ventricle and cardiac output [115]. A clinical trial including patients with chronic ischemic cardiomyopathy reported that administering SVF induced better heart function and better performance in walk tests [116]. Implantation of ASCs seeded in biomaterials was also found to accelerate would healing by providing better neovascularization and up-regulating expression of dermal tissue components [117].

The immunomodulatory potential of ASCs has been effective for autoimmune diseases, including sclerosis, arthritis, and type 1 diabetes. Intravenous administration of ASCs was reported to reduce severity of experimental autoimmune encephalomyelitis by reducing tissue damage, infiltration of immune cells, and serum levels of pro-inflammatory cytokines [118,119]. In clinical studies on patients with knee osteoarthritis, intra-articular injection of ASCs resulted in pain relief and functional improvement without adverse effects [120,121]. In streptozotocin-induced diabetic rats, both human and rat ASCs were shown to differentiate into insulin-producing cells, lower blood glucose levels, and increase glucose sensitivity by suppressing pancreatic islet damage and up-regulating insulin protein [122,123].

The capability of ASCs to secrete neurotrophic mediators could be utilized for treatment of neurodegenerative diseases, such as Alzheimer’s, Parkinson’s, and amyotrophic lateral sclerosis (ALS). Transplantation of ASCs down-regulated expression of amyloid-β (Aβ), crucially involved in the development of Alzheimer’s disease, and up-regulated Aβ-degrading enzymes in an Alzheimer’s disease mouse model [124]. In another study, it increased secretion of anti-inflammatory cytokines, which induces polarization of microglia toward the alternatively activated phenotype, and reversed learning and memory function [125]. ASC treatment was also shown to increase leptin secretion, which promotes neurogenesis and suppresses neurodegeneration in an Alzheimer’s disease model [126]. Intravenous injection of human ASCs to a Parkinson’s mouse model significantly improved behavioral performances, rescued dopaminergic neurons, and restored mitochondrial activity [127]. Moreover, ASC injection into an ALS mouse model up-regulated expression of metallothionein and activity of glutathione S-transferase, which is closely related to progression of the diseases, and improved survival and motor functions [128]. The injection of ASCs was also shown to significantly delay motor deterioration in mice with ALS by modulating protein secretion from local glial cells toward a neuroprotective phenotype [129].

ASC therapy were also shown to alleviate obesity-induced metabolic disorders. In diabetic mice induced by HFD feeding, systemic ASC transplantation ameliorated metabolic dysregulation by supporting pancreatic islet growth, reducing fat accumulation in the liver, increasing GLUT4 expression in skeletal muscle, and suppressing macrophage infiltration in adipose tissue [130]. Another study using HFD-fed mice reported that intravenous injection of ASCs down-regulated PPARγ, IL-6, and F4/80 expression in the liver and F4/80 and TNF-α expression in the pancreas, which suppressed fatty liver development and preserved pancreatic β-cell mass. The treatment also reduced serum TAG and glucose levels and increased serum HDL level [131].

Taken together, emerging evidence demonstrates the therapeutic potential of ASCs in combating multiple types of diseases. Although numerous *in vivo* and *in vitro* studies have been conducted and have provided promising data, more pre-clinical and clinical trials are still required for clinical application of ASCs. Determination of effective timing of ASC treatment, appropriate methods of delivery, and optimal dose and intervals will substantially contribute to develop new, safe, and efficient therapeutic strategies.

### Beige adipocytes as a potential anti-obesity therapy

Beige adipocytes have been the subject of intense focus as a cellular target for anti-obesity therapy partly because: (**1**) beige adipocytes appear to be more relevant to adult humans [102,132–134]; (**2**) beige energy burning cells could be largely recruited to convert glucose and fatty acids into heat, whereas brown adipocytes are limited to small deposits and limited to babies; and (**3**) beige adipocytes could be induced throughout WAT by the various ways that have no effect on brown adipocytes [135,136]. Recent studies have shown that adult humans have beiging capacity. Beige adipocytes are generated in cold-exposed humans, which confers metabolic benefit such as reduced blood glucose and increased energy expenditure [137–142]. Also, our findings and those of others have demonstrated that beige adipocytes can reduce metabolic syndrome in both mice and humans [20–24,142–145], raising the possibility of modulating beige adipocytes as a potential anti-obesity therapy.

However, it has not been very successful to apply various molecular cues and stimuli to induce beige adipocyte formation in humans and to modify obesity and diabetes by stimulating UCP1 activity in adipose tissue with drugs. This is because overuse of the stimuli, including β3 adrenergic receptor agonists and PPARγ ligands, has strong side effects and can cause serious health problems, and the potential to form beige adipocytes fails in obese patients or middle aged and older individuals. Therefore, to overcome these clinical hurdles, more studies are needed to identify the events and mechanisms that modulate the development of beige fat.

## Conclusion and future directions

In summary, ASCs play a critical role in adipose tissue development, homeostasis, thermogenesis, and even healthy expansion by generating new adipocytes when faced with excess caloric intake. There is high heterogeneity and plasticity of ASCs among distinct adipose tissue depots and at different phases of adipose tissue development, and obesity induces metabolic dysregulation by altering both number and functions of ASCs in adipose tissue. Although a wealth of knowledge about ASCs and adipose tissue biology has been recently gained, many critical questions regarding the nature of ASCs and their regulations of adipogenesis in health and obesity remain unclear. Since the signals associated with HFD feeding and their effects on ASC function and behavior have not been identified, a better understanding of underlying mechanisms and clinical trials on human ASCs is still sought for. These studies may be of great assistance for the development of therapeutic strategies against obesity-associated metabolic disorders.

## Abbreviations

ALS: amyotrophic lateral sclerosis
APC: adipose progenitor/precursor cell
aP2: adipocyte protein 2
ASC: adipose stem cell
ATM: adipose tissue macrophage
Aβ: amyloid-β
BAT: brown adipose tissue
BMI: body mass index
CD: cluster of differentiation
C/EBP: CCAAT/enhancer binding protein
GLUT: glucose transporter
HDL: high-density lipoprotein
HIF: hypoxia-inducible factor
HFD: high-fat diet
IL: interleukin
iNOS: inducible nitric oxide synthase
LD: lipid droplet
Lysm: lysin motif
MCP-1: monocyte chemoattractant protein-1
*Myf5*: myogenic factor 5 encoding gene
M1: type 1 macrophage
M2: type 2 macrophage
Pax3: paired box gene 3
PDGFR: platelet-derived growth factor receptor
PPARγ: peroxisome proliferator-activated receptor γ
Pref-1: preadipocyte factor 1
Prx-1: paired-related homeobox 1
ROS: reactive oxygen species
SAT: subcutaneous adipose tissue
SM22: smooth muscle protein 22
Sox10: SRY-Box transcription factor 10
SVF: stromal vascular fraction
TAG: triacylglycerol
TNF-α: tumor necrosis factor-α
UCP1: uncoupling protein 1
VAT: visceral adipose tissue
VE: vascular endothelial
WAT: white adipose tissue
WT1: Wilms tumor gene
α-SMA: α-smooth muscle actin
β-AR: β-adrenergic receptor
